# Utility values for specific hepatic encephalopathy health states elicited from the general public in the United Kingdom

**DOI:** 10.1186/1477-7525-12-89

**Published:** 2014-06-10

**Authors:** Julian F Guest, Kam Nanuwa, Rob Barden

**Affiliations:** 1 Catalyst Health Economics Consultants, 34b High Street, Northwood, Middlesex HA6 1BN, UK; 2 School of Biomedical Sciences, King’s College, London, UK; 3 Norgine Pharmaceuticals Ltd, Harefield, UK

**Keywords:** Conn grade, Hepatic encephalopathy, Standard gamble, Time trade-off, Utility, UK

## Abstract

**Background and aims:**

To elicit utility values for five health states corresponding to increasing severity of hepatic encephalopathy, from members of the general public in the UK. The health states studied were Conn grades 0, 1, 2, 3 and 4.

**Methods:**

Interviewer-administered time trade-off (TTO) and standard gamble (SG) utilities were elicited for the five health states from a random sample of 200 members of the general public in the UK, using health state descriptions validated by clinicians and members of the general public.

**Results:**

Respondents’ mean age was 49.5 years and 49% were female. Mean utilities were 0.962 (TTO) and 0.915 (SG) for Conn grade 0; 0.912 (TTO) and 0.837 (SG) for Conn grade 1; 0.828 (TTO) and 0.683 (SG) for Conn grade 2; 0.691 (TTO) and 0.489 (SG) for Conn grade 3; and 0.429 (TTO) and 0.215 (SG) for Conn grade 4. The TTO and SG values between the five Conn grades were significantly different (*p* < 0.001). Additionally, the TTO value was significantly higher than the SG value for the corresponding state (*p* <0.0001).

**Conclusion:**

These findings quantify how different Conn grades and level of response to treatment may impact on the health-related quality of life of patients with hepatic encephalopathy. There were greater preference values for lower levels of disease, with the highest value associated with Conn grade 0. These health state preference values can be used to estimate the outcomes of different interventions for hepatic encephalopathy in terms of quality-adjusted life years.

## Introduction

Hepatic encephalopathy (HE) is a reversible neuropsychiatric disorder caused by an accumulation of toxins in the bloodstream that are normally removed by the liver. HE encompasses a spectrum of neuropsychiatric abnormalities seen in patients with established liver disease, and is most commonly associated with liver cirrhosis [1,2].

HE can be graded using the Conn grade (also called the West Haven classification) in which higher scores indicate higher disease severity. Grade 0 represents no personality or behavioural abnormality detected. Grade 1 represents lack of awareness, euphoria or anxiety, shortened attention span, impaired performance of addition. Grade 2 represents lethargy or apathy, minimal disorientation for time or place, subtle personality change, inappropriate behaviour, impaired performance of subtraction. Grade 3 represents somnolence to semi stupor but responsive to verbal stimuli, confusion, gross disorientation. Grade 4 represents coma (unresponsive to verbal or noxious stimuli) [2-4]. Approximately 70% of patients with liver cirrhosis present with subclinical or mild HE, and 23-40% progress to a more severe form of the disease [5-7].

In 2011, the ISHEN (International Society for Hepatic Encephalopathy and Nitrogen Metabolism) classification system [8] was proposed, but is yet to be fully adopted by the clinical community. The system classifies patients as being “unimpaired” (no clinical neurophysiological/neuropsychometric changes), or having “covert” HE (i.e. minimal HE or HE with a Conn grade of 1), or having “overt” HE (i.e. clinically relevant HE or HE with a Conn grade of 2, 3 or 4). After an overt episode, patients usually return to an unimpaired or minimal HE state and this is considered to constitute a state of remission.

HE is associated with diminished health-related quality of life (HRQoL) across both physical and mental domains [9-14] compared to the general population and is similar to that observed in patients with other chronic diseases, such as congestive heart failure [9]. The impairment in HRQoL, as measured by the Chronic Liver Disease Questionnaires (CLDQ), found that patients who experience overt breakthrough HE have a significantly lower overall HRQoL score than patients who maintain remission from overt HE [14].

Utilities are a measure of an individual’s preference for, or desirability of, a specific level of health status or specific health outcome [15]. Quantifying the subjective impact of treatment on patients, by estimating their utility preference for different health states, is key in comparing the cost-effectiveness of alternative treatments. Estimating patients’ health status utility enables preferences to be quantified for selected clinical outcomes, and life expectancy to be quality-adjusted.

Instruments that are used to measure an individual’s health status can be generic or disease-specific. Moreover, the valuation of health states can be undertaken using a variety of techniques including time trade-off (TTO) and standard gamble (SG) methods. In TTO, responders are given a choice between two health profiles; a particular health state for a given number of years and full health for a shorter period of time. In effect they are asked to trade between quality of life and length of life. The method tries to establish where they are indifferent between the two by varying the amount of time spent in full health. In SG, respondents are given the choice between two alternatives; one being a health state with certainty and the other being a gamble with two possible outcomes that involve two different health states with particular probabilities attached to each of them. The probabilities attached to the health states are varied until the responder is indifferent between the two alternatives. Generally, TTO is widely seen as an acceptable compromise between simplicity and theoretical rigour [16].

Published utilities for the different Conn grade health states were not available and the only available published estimates were for the remission and overt states. One study reported a utility value of 0.60 for the HE health state using SG methodology among a sample of nine patients with decompensated cirrhosis [17,18]. The corresponding value for patients with compensated cirrhosis was 0.80 [18]. The other studies used a panel of hepatologists to elicit utility values for a number of health states for liver disease, including HE [19,20]. Accordingly, this study aimed to elicit preference values for the five individual Conn grade health states from randomly selected members of the general public in the UK using standard gamble (SG) and time trade-off (TTO) techniques.

## Methods

### Health states

Descriptions of five HE health states (i.e. Conn grade 0, Conn grade 1, Conn grade 2, Conn grade 3 and Conn grade 4) were developed in collaboration with clinicians using published literature [4,8,21,22]. Each health state described the typical patient experience across several domains including symptoms, treatment, response, management and prognosis, enabling a balanced description across all five health states. The health states were refined after iterative review by clinicians and piloting the descriptions among a sample of 20 members of the general public in London, UK. Each health state description (Table 1) is a lay interpretation of the original wording in the cited references, in order to paint a meaningful picture of the respective Conn grades to members of the general public.

**Table 1:** Health state descriptions for the five HE health states

**Non-technical description of hepatic encephalopathy**	
➢	The liver is an organ in the abdomen that processes nutrients and fluids and removes toxins and harmful substances from the blood.
➢	Liver cirrhosis is the end result of liver damage caused by alcohol abuse or hepatitis or poor diet or obesity or some bile duct diseases.
➢	Individuals with liver cirrhosis generally present with an enlarged liver and specific blood tests and scans may be required to confirm a liver problem. A liver biopsy may also be required.
➢	Damage caused by liver cirrhosis is permanent and the liver cannot return to normal. Therefore, the aim of treatment is to prevent further damage and manage any complications.
➢	Patients with severe liver cirrhosis can develop hepatic encephalopathy. Symptoms include forgetfulness, confusion, personality changes, problems with muscles and movement of limbs and possibly coma. There are five grades of hepatic encephalopathy and the symptoms affect a patient to varying degrees depending on the grade of disease.
➢	Treatment usually involves medication and changes to diet and lifestyle. If the symptoms cannot be controlled a liver transplant may be required.
➢	An estimated 58% of patients usually die within one year of experiencing an episode of hepatic encephalopathy and 77% have usually died within three years.
**Grade 0**	
➢	Patients can experience tiredness, itching, loss of appetite, nausea, weight loss and bruising of the skin.
➢	Patients may also find it difficult to pay attention and their reaction times may be slower than normal, so it can take longer to complete usual daily activities.
➢	Patients might see their doctor and receive general advice and minimal treatment.
➢	Very few patients who remain in Grade 0 will die.
**Grade 1**	
➢	Patients have the same symptoms as those in Grade 0. However, they may also experience a slight lack of awareness, anxiety, feelings of well-being or happiness.
➢	These patients may also find it harder to pay attention and have difficulty sleeping and performing usual daily activities.
➢	Patients are more likely to see their doctor, who would try to establish the cause of the worsening symptoms, and receive general advice and appropriate medical treatment.
➢	Very few patients who remain in Grade 1 will die.
**Grade 2**	
➢	Patients in Grade 2 have the same symptoms as those in Grade 1. In addition they may feel very tired, lack energy and become forgetful.
➢	These patients will take longer than normal to perform usual daily activities and may become disoriented and detached from family and friends.
➢	Their speech may be slow or slurred and they may not respond to questions as normal.
➢	They may be irritable and more child-like in their behaviour.
➢	Patients may be hospitalised for a few days for tests to try to establish the cause of the worsening symptoms, and receive appropriate supportive care and medical treatment. Afterwards, patients may or may not return to the way they were before.
➢	An estimated 20% of patients in Grade 2 will die.
**Grade 3**	
➢	Patients in Grade 3 have the same symptoms as those in Grade 2. In addition, they may feel very drowsy or sleepy for long periods or confused and feel as if their brain is unable to function as usual.
➢	They may be unable to perform usual daily activities, such as reading and writing, and may be unable to respond if someone speaks to them.
➢	Their body may not be responsive and their speech may be slow or slurred.
➢	Patients will be hospitalised for a few days or a few weeks for tests to try to establish the cause of the worsening symptoms, and receive appropriate supportive care and medical treatment. Afterwards, patients may or may not return to the way they were before.
➢	An estimated 56% of patients in Grade 3 will die.
**Grade 4**	
➢	Patients in Grade 4 will be unconscious in a coma.
➢	These patients will be hospitalised for a few weeks or maybe even months.
➢	They will be given tests to try to establish the cause of the worsening symptoms, and receive appropriate supportive care, including oxygen to help them breathe, as well as medical treatment.
➢	These patients are unable to perform any daily activities while they are in a coma.
➢	Patients who survive may or may not return to the way they were before.
➢	An estimated 75% of patients in Grade 4 will die.

### Study respondents

A sample of 267 randomly selected members of the general public at six different locations across Greater London, UK were invited to participate in the study. Of these, 67 subjects declined. Hence, the study was undertaken among a sample of 200 randomly selected members of the general public using non-probability sampling. The interviewers were unaware whether the individuals contacted had any family or friends suffering from HE. Respondents had to be aged between 35 and 65 years of age with or without any liver disease. Potential respondents were excluded if they were not English-speaking, or if they had apparent cognitive impairment, or if in the interviewers' opinion they were incapable of understanding the task. Recruitment occurred during January 2013 and none of the respondents received any remuneration for their participation.

### Data collection and analysis

Data were collected through individual, face-to-face interviews, which were conducted using an interview script. At the start of the interview the nature of the questionnaire was explained, after which participants were asked a range of socio-demographic questions about themselves. Participants were then asked to read a short, non-technical description of HE and the five different health states. Afterwards, they were asked what proportion of their remaining lifetime they would be willing to sacrifice in return for not living with the symptoms associated with each of the five health states being evaluated. The TTO approach was open-ended and the interviewers did not use any props.

While the aim of the study was to elicit preference values using the TTO approach, values were also elicited using the SG approach. This involved asking participants to choose between the certainty of living with the symptoms associated with each health state or gambling on a treatment with two possible outcomes: successful treatment or death. The search procedure used in the SG approach was simple titration and the interviewers used diagrams to help respondents visualise the trade-offs involved. No other props were used. Participants were also asked to rate their current health on a horizontal visual analogue scale (range, 0 to 1).

Using the methodology described by Hammerschmidt *et al*. [23], utility values (ranging from 1.0 for perfect health to 0 for death) were obtained for the five different health states. Differences between groups were tested for statistical significance using analysis of variance (ANOVA) comparing pair-wise means at the *p* < 0.05 level and Tukey’s honestly significant difference (HSD) multiple comparison post-hoc analysis to determine which means were similar and which were different.

The preference values associated with the five different health states were stratified by baseline variables including age, gender, marital status, employment status, annual income, preference value for their current health, whether they had any illness or knew someone with liver disease. Linear regression was performed to assess the relationship between all these independent variables and the respondents’ preference values for each Conn grade.

## Results

The study sample comprised 200 participants who were interviewed in Greater London. The respondents’ mean age was 49.5 (95% CI: 48.1; 50.9) years and 49% were female. Three percent of respondents had an illness at the time of the interview and 7% knew individuals with liver disease. Overall, the participants rated their current health with a utility value of 0.93 (95% CI: 0.91; 0.94) using a horizontal visual analogue scale (VAS). Linear regression demonstrated that the respondents’ preference value for their current health was not affected by their age, gender, marital status, employment status, annual income, whether they had any illness or knew someone with liver disease.

Using the TTO approach, Conn grade 0 was the most preferred health state, with a mean utility of 0.962 in the overall sample (Table 2). The second-most preferred health state was Conn grade 1 followed by Conn grade 2 and Conn grade 3 (mean utility in the overall sample were 0.912, 0.828 and 0.691 respectively). The least preferred health state was Conn grade 4 with a mean utility of 0.429 in the overall sample. The mean utility scores between the five Conn grades were different (*p* < 0.001, ANOVA; Table 3). However, Tukey’s HSD multiple comparison post-hoc analysis showed that the mean utility score for Conn grades 0 and 1 were not significantly different, but Conn grades 1, 2, 3 and 4 were significantly different from one another (Table 4).

**Table 2:** Mean utilities (95% confidence intervals) for five HE health states

	**Mean utilities (95% confidence intervals) for**				
	**Conn grade 0**	**Conn grade 1**	**Conn grade 2**	**Conn grade 3**	**Conn grade 4**
**Utilities elicited**					
**Using TTO**					
Whole cohort	0.962	0.912	0.828	0.691	0.429
	(0.952; 0.972)	(0.896; 0.929)	(0.803; 0.852)	(0.656; 0.726)	(0.371; 0.487)
**Utilities elicited**					
**Using SG**					
Whole cohort	0.915	0.837	0.683	0.489	0.215
	(0.897; 0.932)	(0.816; 0.858)	(0.657; 0.709)	(0.459; 0.519)	(0.190; 0.240)

**Table 3:** Analysis of variance (ANOVA)

	**Sum of squares**	**Degrees of freedom**	**Mean square**	**F value**	**Significance**
**TTO values**					
Between groups	36.567	4	9.142	158.636	0.000
Within groups	57.339	995	0.058		
Total	93.906	999			
**SG values**					
Between groups	63.715	4	15.929	516.367	0.000
Within groups	30.693	995	0.031		
Total	94.408	999			

**Table 4:** Tukey’s HSD multiple comparison post-hoc analysis

**Conn grade (I)**	**Conn grade (J)**	**Mean Difference between I and J**	**Std. Error**	**Significance**	**95% Confidence Interval**	
					**Lower bound**	**Upper bound**
**TTO values**						
0	1	0.049	0.024	0.240	-0.016	0.115
	2	0.134	0.024	0.000	0.069	0.200
	3	0.271	0.024	0.000	0.205	0.337
	4	0.533	0.024	0.000	0.467	0.599
1	0	-0.049	0.024	0.240	-0.115	0.016
	2	0.085	0.024	0.004	0.019	0.150
	3	0.221	0.024	0.000	0.156	0.287
	4	0.484	0.024	0.000	0.418	0.549
2	0	-0.134	0.024	0.000	-0.200	-0.069
	1	-0.085	0.024	0.004	-0.150	-0.019
	3	0.137	0.024	0.000	0.071	0.202
	4	0.399	0.024	0.000	0.333	0.464
3	0	-0.271	0.024	0.000	-0.337	-0.205
	1	-0.221	0.024	0.000	-0.287	-0.156
	2	-0.137	0.024	0.000	-0.202	-0.071
	4	0.262	0.024	0.000	0.196	0.328
4	0	-0.533	0.024	0.000	-0.599	-0.467
	1	-0.484	0.024	0.000	-0.549	-0.418
	2	-0.399	0.024	0.000	-0.464	-0.333
	3	-0.262	0.024	0.000	-0.328	-0.196
**SG values**						
0	1	0.078	0.018	0.000	0.030	0.126
	2	0.232	0.018	0.000	0.184	0.280
	3	0.426	0.018	0.000	0.378	0.474
	4	0.700	0.018	0.000	0.652	0.748
1	0	-0.078	0.018	0.000	-0.126	-0.030
	2	0.154	0.018	0.000	0.106	0.202
	3	0.348	0.018	0.000	0.300	0.396
	4	0.622	0.018	0.000	0.574	0.670
2	0	-0.232	0.018	0.000	-0.280	-0.184
	1	-0.154	0.018	0.000	-0.202	-0.106
	3	0.194	0.018	0.000	0.146	0.242
	4	0.468	0.018	0.000	0.420	0.516
3	0	-0.426	0.018	0.000	-0.474	-0.378
	1	-0.348	0.018	0.000	-0.396	-0.300
	2	-0.194	0.018	0.000	-0.242	-0.146
	4	0.274	0.018	0.000	0.226	0.322
4	0	-0.700	0.018	0.000	-0.748	-0.652
	1	-0.622	0.018	0.000	-0.670	-0.574
	2	-0.468	0.018	0.000	-0.516	-0.420
	3	-0.274	0.018	0.000	-0.322	-0.226

Similar trends were observed using SG (Tables 2 and 3), although the utility values were all significantly lower than those derived by TTO (*p* <0.001). Additionally, the utility values for each state were significantly different from one another (*p* <0.001; Table 4)

Mean utility values elicited from respondents, stratified by their gender, age, whether they knew someone with liver disease, marital status, employment status and annual income is shown in Tables 5 and 6.

**Table 5:** Mean utilities (95% confidence intervals) for five HE health states, stratified by socio-demographic parameters, using TTO

	**Mean utilities (95% confidence intervals) for**				
	**Conn grade 0**	**Conn grade 1**	**Conn grade 2**	**Conn grade 3**	**Conn grade 4**
**Respondents’ gender**					
Male (n = 101)	0.97 (0.96; 0.98)	0.93 (0.91; 0.95)	0.84 (0.80; 0.87)	0.69 (0.65; 0.74)	0.45 (0.38; 0.53)
Female (n = 99)	0.95 (0.93; 0.97)	0.90 (0.87; 0.92)	0.82 (0.79; 0.85)	0.69 (0.64; 0.74)	0.41 (0.32; 0.49)
**Respondents’ age**					
30-39 years (n = 44)	0.95 (0.92; 0.97)	0.90 (0.87; 0.94)	0.83 (0.78; 0.88)	0.75 (0.70; 0.81)	0.58 (0.50; 0.66)
40-49 years (n = 43)	0.96 (0.94; 0.98)	0.90 (0.87; 0.93)	0.82 (0.78; 0.86)	0.69 (0.63; 0.75)	0.45 (0.32; 0.58)
50-59 years (n = 79)	0.97 (0.95; 0.98)	0.92 (0.89; 0.95)	0.81 (0.77; 0.85)	0.65 (0.58; 0.71)	0.34 (0.24; 0.45)
≥60 years (n = 34)	0.98 (0.95; 1.00)	0.93 (0.89; 0.98)	0.87 (0.80; 0.94)	0.63 (0.57; 0.81)	0.32 (0.23; 0.55)
**Respondents had an illness**					
No (n = 194)	0.96 (0.95; 0.97)	0.91 (0.89; 0.93)	0.83 (0.80; 0.85)	0.69 (0.66; 0.73)	0.43 (0.37; 0.49)
Yes (n = 6)	0.97 (0.92; 1.00)	0.92 (0.83; 1.00)	0.85 (0.71; 0.99)	0.66 (0.47; 0.85)	0.45 (0.18; 0.72)
**Respondent knew someone with liver disease**					
No (n = 187)	0.96 (0.95; 0.97)	0.91 (0.90; 0.93)	0.83 (0.81; 0.86)	0.69 (0.66; 0.73)	0.44 (0.38; 0.50)
Yes (n = 13)	0.95 (0.91; 0.99)	0.88 (0.82; 0.93)	0.78 (0.71; 0.85)	0.66 (0.53; 0.78)	0.28 (0.09; 0.46)
**Respondents’ marital status**					
Married (n = 98)	0.98 (0.97; 0.99)	0.94 (0.92; 0.95)	0.85 (0.82; 0.89)	0.72 (0.67; 0.76)	0.45 (0.38; 0.53)
Co-habiting (n = 33)	0.95 (0.92; 0.98)	0.89 (0.85; 0.93)	0.79 (0.73; 0.85)	0.67 (0.59; 0.75)	0.47 (0.35; 0.59)
Single (n = 31)	0.95 (0.92; 0.98)	0.91 (0.85; 0.96)	0.83 (0.77; 0.88)	0.74 (0.66; 0.81)	0.47 (0.28; 0.65)
Divorced/separated (n = 37)	0.94 (0.91; 0.97)	0.87 (0.82; 0.93)	0.78 (0.71; 0.86)	0.60 (0.49; 0.70)	0.31 (0.15; 0.47)
Widowed (n = 1)	1.00	1.00	1.00	1.00	0.23
**Respondents’ employment status**					
Employed full-time (n = 135)	0.96 (0.95; 0.98)	0.91 (0.89; 0.93)	0.82 (0.79; 0.85)	0.67 (0.63; 0.71)	0.43 (0.36; 0.49)
Self-employed (n = 23)	0.98 (0.96; 1.00)	0.96 (0.93; 0.99)	0.87 (0.82; 0.92)	0.74 (0.66; 0.82)	0.49 (0.36; 0.63)
Employed part-time (n = 21)	0.95 (0.91; 0.99)	0.91 (0.84; 0.98)	0.86 (0.76; 0.96)	0.70 (0.56; 0.84)	0.39 (0.18; 0.59)
Retired (n = 6)	0.99 (0.97; 1.00)	0.98 (0.95; 1.00)	0.98 (0.95; 1.00)	0.92 (0.82; 1.00)	0.70 (0.60; 0.81)
Unemployed (n = 4)	0.94 (0.86; 1.00)	0.93 (0.85; 1.00)	0.84 (0.71; 0.97)	0.71 (0.43; 0.98)	0.40 (0.09; 0.72)
Student (n = 11)	0.91 (0.84; 0.99)	0.82 (0.71; 0.92)	0.72 (0.62; 0.83)	0.65 (0.49; 0.81)	0.26 (0.00; 0.70)
Respondents’ annual income*					
<£15000 (n = 13)	0.95 (0.89; 1.00)	0.92 (0.82; 1.00)	0.89 (0.79; 0.98)	0.83 (0.72; 0.93)	0.48 (0.10; 0.85)
£15,000-£25,000 (n = 20)	0.97 (0.94; 0.99)	0.91 (0.87; 0.95)	0.86 (0.79; 0.92)	0.78 (0.69; 0.87)	0.47 (0.30; 0.64)
£25,001-£35,000 (n = 89)	0.96 (0.94; 0.97)	0.90 (0.88; 0.93)	0.81 (0.78; 0.85)	0.66 (0.61; 0.71)	0.43 (0.35; 0.51)
>£35,000 (n = 75)	0.97 (0.95; 0.99)	0.92 (0.90; 0.95)	0.83 (0.79; 0.87)	0.68 (0.62; 0.74)	0.41 (0.31; 0.51)

*3 respondents declined to provide their annual income.

**Table 6:** Mean utilities (95% confidence intervals) for five HE health states, stratified by socio-demographic parameters, using SG

	**Mean utilities (95% confidence intervals) for**				
	**Conn grade 0**	**Conn grade 1**	**Conn grade 2**	**Conn grade 3**	**Conn grade 4**
**Respondents’ gender**					
Male (n = 101)	0.93 (0.90; 0.95)	0.86 (0.83; 0.89)	0.70 (0.67; 0.74)	0.51 (0.47; 0.56)	0.23 (0.20; 0.27)
Female (n = 99)	0.90 (0.87; 0.93)	0.82 (0.79; 0.85)	0.66 (0.63; 0.70)	0.46 (0.42; 0.51)	0.19 (0.16; 0.23)
**Respondents’ age**					
30-39 years (n = 44)	0.88 (0.82; 0.94)	0.81 (0.74; 0.87)	0.69 (0.62; 0.76)	0.50 (0.43; 0.57)	0.20 (0.15; 0.26)
40-49 years (n = 43)	0.91 (0.88; 0.94)	0.83 (0.79; 0.87)	0.65 (0.60; 0.70)	0.45 (0.39; 0.51)	0.19 (0.14; 0.24)
50-59 years (n = 79)	0.93 (0.91; 0.94)	0.84 (0.81; 0.87)	0.68 (0.64; 0.72)	0.48 (0.43; 0.52)	0.21 (0.17; 0.25)
≥60 years (n = 34)	0.93 (0.90; 0.97)	0.88 (0.84; 0.92)	0.72 (0.66; 0.77)	0.55 (0.48; 0.62)	0.27 (0.21; 0.33)
**Respondents had an illness**					
No (n = 194)	0.91 (0.90; 0.93)	0.83 (0.81; 0.86)	0.68 (0.65; 0.71)	0.48 (0.45; 0.51)	0.21 (0.18; 0.23)
Yes (n = 6)	0.90 (0.81; 0.99)	0.90 (0.85; 0.95)	0.80 (0.71; 0.89)	0.63 (0.55; 0.72)	0.43 (0.30; 0.56)
**Respondent knew someone with liver disease**					
No (n = 187)	0.91 (0.89; 0.93)	0.83 (0.81; 0.86)	0.68 (0.65; 0.71)	0.48 (0.45; 0.51)	0.21 (0.19; 0.24)
Yes (n = 13)	0.93 (0.90; 0.97)	0.87 (0.81; 0.93)	0.75 (0.68; 0.83)	0.58 (0.49; 0.68)	0.27 (0.17; 0.36)
**Respondents’ marital status**					
Married (n = 98)	0.93 (0.91; 0.95)	0.85 (0.83; 0.88)	0.70 (0.67; 0.74)	0.52 (0.48; 0.56)	0.24 (0.20; 0.27)
Co-habiting (n = 33)	0.89 (0.83; 0.95)	0.81 (0.74; 0.87)	0.64 (0.57; 0.71)	0.44 (0.36; 0.52)	0.19 (0.13; 0.25)
Single (n = 31)	0.89 (0.82; 0.96)	0.85 (0.77; 0.92)	0.73 (0.64; 0.81)	0.54 (0.46; 0.62)	0.22 (0.14; 0.30)
Divorced/separated (n = 37)	0.91 (0.88; 0.94)	0.82 (0.78; 0.86)	0.64 (0.58; 0.70)	0.42 (0.36; 0.47)	0.18 (0.13; 0.22)
Widowed (n = 1)	0.90	0.70	0.40	0.20	0.10
**Respondents’ employment status**					
Employed full-time (n = 135)	0.92 (0.91; 0.94)	0.84 (0.82; 0.86)	0.69 (0.66; 0.72)	0.49 (0.46; 0.53)	0.22 (0.19; 0.25)
Self-employed (n = 23)	0.87 (0.78; 0.96)	0.81 (0.72; 0.90)	0.67 (0.57; 0.77)	0.47 (0.38; 0.56)	0.20 (0.11; 0.30)
Employed part-time (n = 21)	0.93 (0.88; 0.98)	0.83 (0.77; 0.88)	0.66 (0.58; 0.74)	0.48 (0.37; 0.59)	0.18 (0.11; 0.25)
Retired (n = 6)	0.95 (0.88; 1.00)	0.90 (0.81; 0.99)	0.75 (0.58; 0.92)	0.60 (0.47; 0.73)	0.32 (0.18; 0.45)
Unemployed (n = 4)	0.88 (0.83; 0.92)	0.75 (0.62; 0.88)	0.55 (0.35; 0.75)	0.38 (0.23; 0.52)	0.25 (0.08; 0.42)
Student (n = 11)	0.85 (0.67; 1.00)	0.81 (0.64; 0.98)	0.70 (0.55; 0.85)	0.52 (0.38; 0.66)	0.17 (0.08; 0.26)
Respondents’ annual income*					
<£15000 (n = 13)	0.85 (0.69; 1.00)	0.80 (0.65; 0.95)	0.66 (0.51; 0.82)	0.55 (0.43; 0.68)	0.19 (0.10; 0.29)
£15,000-£25,000 (n = 20)	0.89 (0.79; 0.99)	0.83 (0.73; 0.93)	0.73 (0.62; 0.84)	0.54 (0.41; 0.66)	0.23 (0.13; 0.33)
£25,001-£35,000 (n = 89)	0.92 (0.90; 0.94)	0.84 (0.82; 0.87)	0.69 (0.66; 0.73)	0.50 (0.46; 0.55)	0.24 (0.20; 0.28)
>£35,000 (n = 75)	0.93 (0.91; 0.94)	0.84 (0.82; 0.87)	0.67 (0.63; 0.71)	0.45 (0.41; 0.50)	0.19 (0.15; 0.22)

*3 respondents declined to provide their annual income.

Linear regression demonstrated that respondents’ gender, annual income, preference value for their current health, whether they had any illness or knew someone with liver disease did not affect their TTO preference values as the significance value was >0.05. When these independent variables were excluded from the regression model it was found that the TTO values elicited from divorced/separated respondents were ~0.07 lower than those elicited from single, married, cohabiting and widowed respondents (*p* <0.05). Additionally, the values elicited from retired respondents were ~0.41 higher than those elicited from those who were employed, unemployed and students (*p* <0.02). Respondents’ age influenced their preference value for Conn grades 3 and 4; the elicited values were reduced by 0.01 for each year of age (*p* < 0.02). Preference values for Conn grades 0, 1 and 2 were unaffected by respondents’ age. Linear regression also showed that respondents’ preference values for any of the health states elicited by SG were not affected by their age, gender, marital status, employment status, annual income, preference value for their current health or whether they knew someone with liver disease. When these independent variables were excluded from the regression model the respondents’ SG preference values for Conn grade 4 were found to be influenced by whether they had any illness. The values elicited by those with any illness were ~0.2 higher than those elicited from the other respondents (*p* = 0.02).

## Discussion

Patients in remission from HE have an underlying risk of experiencing breakthrough overt HE episodes. Moreover, if not prevented, episodes of HE can reoccur and progress in severity. Therefore, over the course of the condition, a patient’s HRQoL is likely to decline at any time, depending on the patient’s underlying risk of experiencing a breakthrough overt HE episode. This study will enable the decline in a patient’s HRQoL to be quantified. Additionally, the preference values elicited in this study will enable a direct comparison between different levels of treatment response to HE, and a more accurate calculation of quality-adjusted life years (QALYs) in economic evaluations of HE treatments.

The mean baseline health status of our sample derived using a VAS was 0.93. Only 3% of our sample had an illness at the time of the interviews. Hence, the health status of our sample was consistent with that reported in the published literature for subjects with a mean age of 48.9 years with no health condition. The mean EQ-5D score was reported to be 0.946 for those with no health condition and 0.843 for those of a similar age irrespective of health status [24]. Utilities were also elicited from our sample using a VAS for the five health states being studied and found to be 0.75 for Conn grade 0, 0.58 for Conn grade 1, 0.40 for Conn grade 2, 0.20 for Conn grade 3 and 0.09 for Conn grade 4. These values were lower than the utilities elicited by TTO and SG. This is consistent with the findings of others who, after a review of utilities across 995 chronic and acute health states, found a strong tendency for VAS to yield lower values than SG and TTO [25].

The utility values obtained using SG were different from those obtained using TTO. Such differences are expected because values elicited by SG are likely to be affected by the respondents’ attitudes toward risk. This is consistent with our finding that respondents’ preference values elicited by SG were influenced by whether they had an illness at the time of the interview. Conversely, values elicited by TTO are likely to be affected by respondents’ time preferences [16]. This is consistent with our finding that respondents’ preference values elicited by TTO were influenced by time-dependent variables. The TTO preference values for the two most critical health states (i.e. Conn grades 3 and 4) were influenced by their age. They were also influenced by whether the respondents were retired. Additionally, differences in utility values between Conn grades elicited by TTO are likely to be smaller than the differences between Conn grades elicited by SG. Hence, it is likely that cost per QALY values that are estimated using utilities elicited by TTO will be larger than corresponding values that are estimated using utilities elicited by SG.

This study was subject to a number of limitations. Firstly, interviews with patients were not included as part of the health state development process for practical reasons, although feedback from patients would have facilitated validation of the health state descriptions. Instead, the study relied on contributions from clinicians, based on the West Haven Classification, published literature and clinical experience, and piloting among members of the general public to validate the health states descriptions. Undoubtedly, validating utility estimates among a population of patients with HE in the future may be advantageous. Secondly, as required by the TTO approach, it was assumed that the relationship between the duration of living in a health state and an individual’s utility value for that health state was independent. This assumption may not be valid since a study using EQ-5D health states found that preferences decline with increasing duration of remaining in a severe health state [26]. Further studies are required to assess whether this assumption is valid for health state valuations in HE using a TTO approach and whether the elicited utility values overestimate true preferences for the five health states. Thirdly, a non-probability sampling technique was used to interview a target population of 200 subjects from across Greater London. We would have preferred to employ a probabilistic sampling method. However, due to time constraints it was not feasible or practical to undertake random sampling. Hence, a purposive, non-probability sampling technique was adopted. The advantage of this sampling method was that a sufficient number of subjects could be interviewed in the limited time available. However, the subjects who participated in this study may not be sufficiently representative of the target population of the UK as a whole. Notwithstanding this, some of our previous utility elicitation studies have been conducted in up to eight different locations across the UK and preference values did not vary across the country [27,28]. Moreover, we have found in previous studies that a minimum of 165 subjects per group is sufficient to detect differences at the <0.05 significance level in TTO and SG values between different health states, if they exist.

Notwithstanding these limitations, this is the first study to estimate preference values for the different Conn grades of HE. The only published cost-effectiveness analysis of competing therapies for HE used a utility value of 0.6 elicited using SG derived from patients with complicated cirrhosis as a proxy for overt HE [17]. Other reported utility values for liver disease are those for patients with end-stage disease awaiting liver transplantation in the US, who have an estimated utility of 0.67 and 0.57 elicited using TTO and SG respectively [29]. In another study using the Health Utility Index 2, US patients with chronic liver disease with and without cirrhosis were reported to have a utility of 0.72 and 0.85 respectively [30]. Conversely, the utility of Iranian patients with decompensated cirrhosis was estimated to be 0.55 elicited using SG [31]. In a systematic review of health state utilities for liver disease, patients with hepatitis C virus who had decompensated cirrhosis were estimated to have a utility of 0.79 elicited using TTO, which was 0.19 higher than utility scores elicited using a VAS [32]. Clearly there is variation in utility values for different health states associated with liver disease. Hence, further research should consider estimating utility values for the different Conn grades from patients with HE using a probabilistic sampling method.

In conclusion, this study indicates greater preference values for lower levels of disease, with the highest value associated with Conn grade 0. Further, this study demonstrates that deeper levels of treatment responses may impact to a greater extent on the health-related quality of life of patients with HE. These health state preference values can be used to estimate the outcomes of interventions for HE in terms of quality-adjusted life years.

## Competing interest

KN and RB are both employees of Norgine Pharmaceuticals. However, the authors have no other competing interest that are directly relevant to the content of this manuscript, which remains their sole responsibility.

## Authors’ contributions

JG designed the study, conducted the analyses and wrote the manuscript. KN and RB scrutinised the analyses and helped interpret some of the findings and edited the manuscript. As lead author I affirm that the manuscript is an honest, accurate and transparent account of the study being reported; that no important aspects of the study have been omitted; and that any discrepancies from the study as planned have been explained. Prof Julian Guest. All authors read and approved the final manuscript.
